# Immunomodulating functions of human leukocyte antigen-G and its role in graft-versus-host disease after allogeneic hematopoietic stem cell transplantation

**DOI:** 10.1007/s00277-021-04486-z

**Published:** 2021-03-11

**Authors:** Xiaoyin Bu, Jinman Zhong, Weiru Li, Shengchun Cai, Ya Gao, Baohong Ping

**Affiliations:** 1 Department of Hematology, Nanfang Hospital, Southern Medical University, Guangzhou, 510515 Guangdong Province China; 2 Department of Huiqiao, Nanfang Hospital, Southern Medical University, Guangzhou, 510515 Guangdong Province China

**Keywords:** Human leukocyte antigen-G, Immune regulation, Transplantation tolerance, Graft-versus-host disease, Allogeneic hematopoietic stem cell transplantation

## Abstract

Allogeneic hematopoietic stem cell transplantation (allo-HSCT) is a potentially curative therapeutic strategy to treat several hematological malignancies and non-hematological malignancies. However, graft-versus-host disease (GVHD) is a frequent and serious transplant-related complication which dramatically restrains the curative effect of allo-HSCT and a significant cause of morbidity and mortality in allogeneic HCT recipients. Effective prevention of GVHD mainly depends on the induction of peripheral immune tolerance. Human leukocyte antigen-G (HLA-G) is a non-classical MHC class I molecule with a strong immunosuppressive function, which plays a prominent role in immune tolerance. HLA-G triggers different reactions depending on the activation state of the immune cells and system. It also exerts a long-term immune tolerance mechanism by inducing regulatory cells. In this present review, we demonstrate the immunomodulatory properties of human leukocyte antigen-G and highlight the role of HLA-G as an immune regulator of GVHD. Furthermore, HLA-G could also serve as a good predictor of GVHD and represent a new therapeutic target for GVHD.

## Introduction

Allogeneic hematopoietic stem cell transplantation has been widely used in the treatment of various hematological malignancies and non-hematological malignancies, which is one of the most effective treatment options to cure these diseases. Despite the continuous improvement of allo-HSCT, the curative effects and broader application of allo-HSCT are still restricted by graft-versus-host disease, which is an inherent treatment-associated adverse immunologic and life-threatening complication after allo-HSCT, with the incidence up to 40–60% and the mortality approaching 15% [1], becoming a major obstacle to transplantation. During HSCT, conditioning regimen (chemotherapy and/or radiotherapy) could lead to the damage and activation of host tissues and then activate host antigen-presenting cells (APCs) which present host antigens to donor effective T cells, leading to allo-activation and inflammatory cytokine release [2].

Human leukocyte antigen-G (HLA-G) is a functional non-classical human leukocyte antigen class Ib molecule [3]. It is widely acknowledged that HLA-G can interact with inhibitory receptors on T cells, B cells, natural killer (NK) cells, as well as antigen-presenting cells (APCs) and can induce the production of tolerant dendritic cells (tDCs) and regulatory T cells (Tregs), so as to inhibit cellular and humoral immune function and thereby induce immune tolerance [3–7]. HLA-G has attracted an extensive research in the fields of immunology and allogenic hematopoietic stem cell transplantation, in the hope that HLA-G could be used as a diagnostic and/or prognostic marker and potential therapy strategies [8–12].

This review summarized the post-transplantation immune tolerance mediated by HLA-G in GVHD, discussed the role of HLA-G in GVHD, and finally reviewed the therapeutic use of HLA-G in GVHD.

## HLA-G molecules

Human leukocyte antigen-G (HLA-G) belongs to the non-classical human major histocompatibility complex (MHC) class Ib whose gene is located on human chromosome 6p21.3, structurally similar to the classical MHC-I antigen. HLA-G gene is composed of 8 exons separated by 7 introns and selectively cleaved during transcription to produce 7 mature mRNA subtypes, encoding 7 protein isoforms, namely, 4 membrane-bound proteins (HLA-G1, G2, G3, and G4) and 3 soluble proteins (HLA-G5, G6, and G7) [13]. With α1, α2, and α3 domains simultaneously, HLA-G1 and HLA-G5 have the ability to combine with beta-2-microglobulin (β2m) to form HLA-G complexes, with the result that they have biological functions and are the main immunomodulatory subtype in the HLA-G family [3, 7, 14].

Physiologically, mRNA of HLA-G transcripts is expressed in most cells and tissues, but mature HLA-G has a restricted tissue expression, which is only expressed at a high level in fetal tissues such as the maternal-fetal interface of trophoblast cells, amniotic cells, and erythroid precursor cells, as well as some adult immunity-exempt tissues such as cornea, thymus, and islet cells [3, 15]. It is also expressed in human peripheral blood mononuclear cell and plasma [16, 17]. Pathological conditions such as viral infections, inflammatory diseases, autoimmune diseases, tumors, and allotransplantation can induce the expression of HLA-G [18].

Numerous studies have demonstrated that HLA-G is a very important immune tolerance molecule. It can maintain maternal-fetal immune tolerance at the maternal-fetal interface, reduce inflammation and immune responses, facilitate tumor immune escape, and strengthen transplantation tolerance [7, 19]. Additionally, HLA-G binds to specific receptors to regulate a variety of immune responses and is categorized as a potential “immune checkpoint” molecule [7].

## HLA-G mediates immune regulation

### Direct regulations of HLA-G on immune cells

#### Effect of HLA-G on T cells

Both donor CD4+ and donor CD8+ T cells have crucial roles in the pathogenesis of GVHD [20]. Acute GVHD involves alloreactive donor T cell–mediated cytotoxic responses against the tissues of the recipient [20, 21]. Proportions of naive cells maturing along Tregs, Th1, Th2, or Th17 phenotypes have influence on the manifestations and severity of GVHD [2].

CD4+ T cells participate in immune regulation and induce immune tolerance through a variety of immunosuppressive mechanisms, which are the key regulator of the autoimmune response and the inflammatory response. HLA-G interacts with ILT-2 receptors expressed on the surface of CD4+T cells, transduces inhibitory signals, prevents the migration of CD4+T cells from G1 phase to G2/M phase, and inhibits the proliferation of CD4+T cells, leading CD4+T cells losing their function, thereby inhibiting the function of most T cells. At the same time, it could also induce the transition of cytokine spectrum to Th2 type, producing cytokines such as interleukin (IL)-10 and other cytokines [7, 22]. In addition, soluble HLA-G (sHLA-G) binding to ILT-2 can dramatically downregulate the expression of chemotactic receptors (such as CCR2, CXCR3, and CXCR5) on CD4+T cells and thus CD4+T cells chemotaxis towards CCL2, CCL8, and CXCL10, and CXCL11 is dampened by sHLA-G [23]. CD4+HLA-G+T cells is a novel subset of CD4+Treg cells in human peripheral blood and inflammatory sites. Compared with CD4+CD25+FOXP3+T regulatory cells, CD4+HLA-G+T cells is capable of secreting high levels of soluble HLA-G5 to inhibit the proliferation of allogeneic T cells, secrete higher levels of IL-10, IL-35, and transforming growth factor (TGF)-β1 and other inhibitory factors, and regulate acquired immune response in vivo. It can substantially reduce the severity of acute graft-versus-host disease in humanized mouse model [24, 25].

CD8+T cells are the most toxic killer cells in T cells, which could differentiate into cytotoxic T lymphocytes (CTL) after activation, in turn causing endothelial damage both in the skin and gut, linked to GVHD [12]. HLA-G can inhibit the killing function of alloantigen-specific CTLs by binding to ILT-2 on the surface of CD8+T cells. Similar to classical MHC-I molecules, HLA-G1 and HLA-G5 have the ability to interact with CD8 co-receptor on T cell subsets, activating the Fas/Fas-Ligand-mediated apoptosis in antigen-specific CD8+T lymphocytes [26, 27]. Besides, the expression of CXCR3 on CD8+T cells are notably downregulated by sHLA-G so as to dampen the chemotaxis of CD8+T cells towards CXCL10 and CXCL11, which are the two ligands of CXCR3 [23].

On the other hand, HLA-G can also influence the secretion of cytokines by helper T cells (Th). HLA-G can increase the secretion of Th2 type cytokines such as IL-3, IL-4, and IL-10 while decreasing the secretion of Th1 type cytokines such as interferon (IFN)-γ and tumor necrosis factor (TNF)-α, causing the immune balance shift to Th2 [16]. At the same time, the cytokines secreted by Th2, such as IL-10, can upregulate the expression of HLA-G, so that a positive feedback reaction and a humoral immune response between HLA-G and IL-10 are formed, resulting in a comprehensive and lasting inhibitory effect [16].

#### Effect of HLA-G on NK cells

Natural killer cells are important effector cells of natural immunity [28]. They exert a killing effect by directly dissolving and secreting cytokines and perform a pivotal role in the body’s immune surveillance and immune defense [28, 29]. The inhibitory effect of HLA-G on NK cells has been confirmed by extensive studies. In vitro studies have proved that HLA-G presented by target cells (cytotrophoblast cells, tumor cells, transfected cell lines) or soluble HLA-G5 present in microenvironment can inhibit the occurrence of cellular immune synapses of NK cells by binding to ILT2 on the surface of them, thus inhibiting the cytolysis of NK cells to target cells [22, 30]. Moreover, HLA-G1 and HLA-G5 have an additive effect on inhibition of NK cell–mediated killing [31]. Additionally, through transferring HLA-G1 and HLA-G3 into human β2m deficient cells, Zhao et al. [32] investigated that β2m-free HLA-G promoted the cytotoxic lysis of NK cells, and meanwhile, the production of proinflammatory cytokines such as IL-1β, TNF-α, and IFN-γ was increased. Morandi et al. [33] revealed that the interaction between soluble HLA-G and ILT2 can downregulate the expression of chemokine receptors (CXCR3 and CD94/NKG2A) on the surface of CD56bright and CD56dim NK cells in peripheral blood and tonsils and, as a result, regulate the chemotaxis of NK cells. Banerjee et al. [34] discovered that the binding of HLA-G to KIR2DL4 on the surface of NK cells does not affect the cytotoxicity of resting NK cells, but can activate NK cells and induce the production of proinflammatory cytokines (such as IFN- γ) and chemokines. However, other studies had found no evidence of functional interaction between sHLA-G and KIR2DL4 in NK cells [35]. Therefore, whether HLA-G/KIR2DL4 interaction affects the activity of NK cells remains controversial [36].

#### Effect of HLA-G on APCs

The activation of antigen-presenting cells plays essential roles in the pathogenesis of GVHD. Dendritic cells (DCs) are the most powerful professional antigen-presenting cells in the body and at the center of initiating, regulating, and maintaining the immune response. Through the release of cytokines, they have the ability to stimulate Th17 responses, thereby initiating and perpetuating GVHD [2]. In vitro, many studies have greatly advanced in the knowledge that HLA-G could inhibit the antigen presentation function of APCs and thus could not effectively stimulate the allogenic proliferation of T cells [7]. HLA-G can also induce ILT4-mediated inhibitory signal, stimulate downstream IL-6/STAT3 signal pathway, downregulate the expression of MHC-II and costimulatory molecules (CD80 and CD86), block antigen presentation, and thus inhibit the maturation and activation of DC cells, inducing DC cells to differentiate into tolerant DC cells, forming immune tolerance [6, 37]. The mouse experimental model also confirmed that HLA-G could inhibit the maturation and activation of immature DC cells into functional mature DC cells [7]. Moreover, HLA-G can also stimulate APCs to secrete cytokines such as TGF- β and IL-10, so that Th1/Th2 balance shifts to Th2.

#### Effect of HLA-G on B cells

During allogeneic hematopoietic stem cell transplantation, the occurrence of cGVHD is partially owing to the production of autoantibodies [2]. Cognate recognition of foreign MHC II of host B cells by donor alloreactive CD4+T cells has been implicated in cGVHD pathogenesis [2]. Naji et al. [38] found that in a microenvironment rich in IL-10 cytokines, HLA-G can interact with ILT2 on the surface of B cells and thus inhibits the proliferation, differentiation, and immunoglobulin secretion of B cells by means of arresting the cell cycle progression at the G0/G1 phase through PKC phosphorylation and AKT dephosphorylation. Beyond that, HLA-G can also downregulate the expression of B cell chemokine receptors (CXCR4 and CXCR5), thereby inhibiting B cell–mediated chemotaxis [38].

### Indirect regulations of HLA-G on immune system

#### Upregulation of inhibitory receptor expression induced by HLA-G

The conducted studies have shown that HLA-G can upregulate the expression of self-inhibitory receptors ILT2, ILT4, and KIR2DL4 on the surface of NK cells, APCs, and CD4+T cells, so as to enhance its own inhibitory effect and exert an indirect immunomodulatory effect [10, 39]. Schwich et al. [40] revealed that sHLA-G1 could significantly increase the ILT2 expression of CD8+T cells, while ILT2 on CD4+T cells was only marginally increased. By upregulation of inhibitory receptors, it is able to enhance the sensitivity of HLA-G to immunosuppression and increase the activation threshold of responding cells. At the same time, it may also be a prerequisite for HLA-G-mediated immunosuppression of cells without HLA-G receptor expression.

#### HLA-G induces the production of regulatory T cells

Regulatory T cells are important immune effector cells to promote and maintain peripheral immune tolerance and normal immune homeostasis and play a critical role in transplantation, autoimmune diseases, infection, tumor, and other diseases. It has been demonstrated that HLA-G is capable to stimulate CD4+T cells and CD8+T cells to differentiate into regulatory T cells, thereby inhibiting the reactivity of other T cells [3]. Selmani et al. [41] confirmed that HLA-G5, in vitro, can induce T cells to differentiate into CD4+CD25highFOXP3+Treg cells and regulate the innate immune response. Regulatory T cells can inhibit the immune activity of CD8+CTL and CD4+T cells through cell-to-cell contact or production of cytokines such as IL-10 and TGF-β. In addition, HLA-G interacts with ILT2/4 on immature or semi-mature DC cells, activates downstream signaling pathways, and induces the production of tolerant DC cells, which in turn induces the secretion of cytokine IL-10 and differentiation of Treg cells and can stimulate T cells anergy and inhibit the proliferation of effector T cells [42, 43].

#### The action mechanism of HLA-G through trogocytosis

Trogocytosis refers to the phenomenon that the cytoplasmic membrane and its related molecules transfer rapidly between cells through cell contact. Through trogocytosis, intact membrane fragments carrying intramembrane and transmembrane proteins can temporarily stay on the surface of the recipient cell, and the cell can temporarily use the proteins it does not express itself and then perform its corresponding function [44]. Studies revealed that through the mechanism of trogocytosis action, T lymphocytes, activated NK cells, and monocytes can obtain HLA-G containing membrane fragments from HLA-G+ antigen-presenting cells or tumor cells. Because the metastatic HLA-G is still functional, T lymphocytes and activated NK cells stop proliferation after receiving the HLA-G membrane fragment and lose their killing ability to the target cells [7]. However, due to the short life span of HLA-G molecules on the surface of monocytes, monocytes do not obtain immunosuppressive function, but can transfer HLA-G membrane fragments obtained from different cell sources together with some of their own membrane fragments to autologous T lymphocytes, thus exerting immune tolerance function [45]. Through the trogocytosis mechanism, the local immunosuppressive effects can occur in the environment where cells expressing HLA-G are scarce.

## HLA-G mediates transplantation tolerance

Graft-versus-host disease is a complicated pathological interaction between the innate and adaptive immune systems of the host and donor, including acute graft-versus-host disease (aGVHD) and chronic graft-versus-host disease (cGVHD). Among them, acute graft-versus-host disease is an immune-mediated process, in which mature donor T cells interact with host and donor antigen-presenting cells, resulting in the release of proinflammatory cytokines, which leads to the activated T cells proliferating and homing to target tissues, eventually causing damage to host tissues. Unlike aGVHD, which is mainly driven by mature donor T lymphocytes, chronic graft-versus-host disease involves more complex immune responses, which are caused by thymus injury, aberrant B cell production, and T cell dysfunction along with cytokine dysregulation [2, 46, 47]. The entire process involves various immune cell subsets (such as host antigen-presenting cells, effector T cells, and natural killer cells), proinflammatory cytokines (such as TNF-α and IL-1), chemokine and chemokine ligands (such as CCR2 and CCR5), and costimulatory molecules [2, 48].

**Fig. 1:**
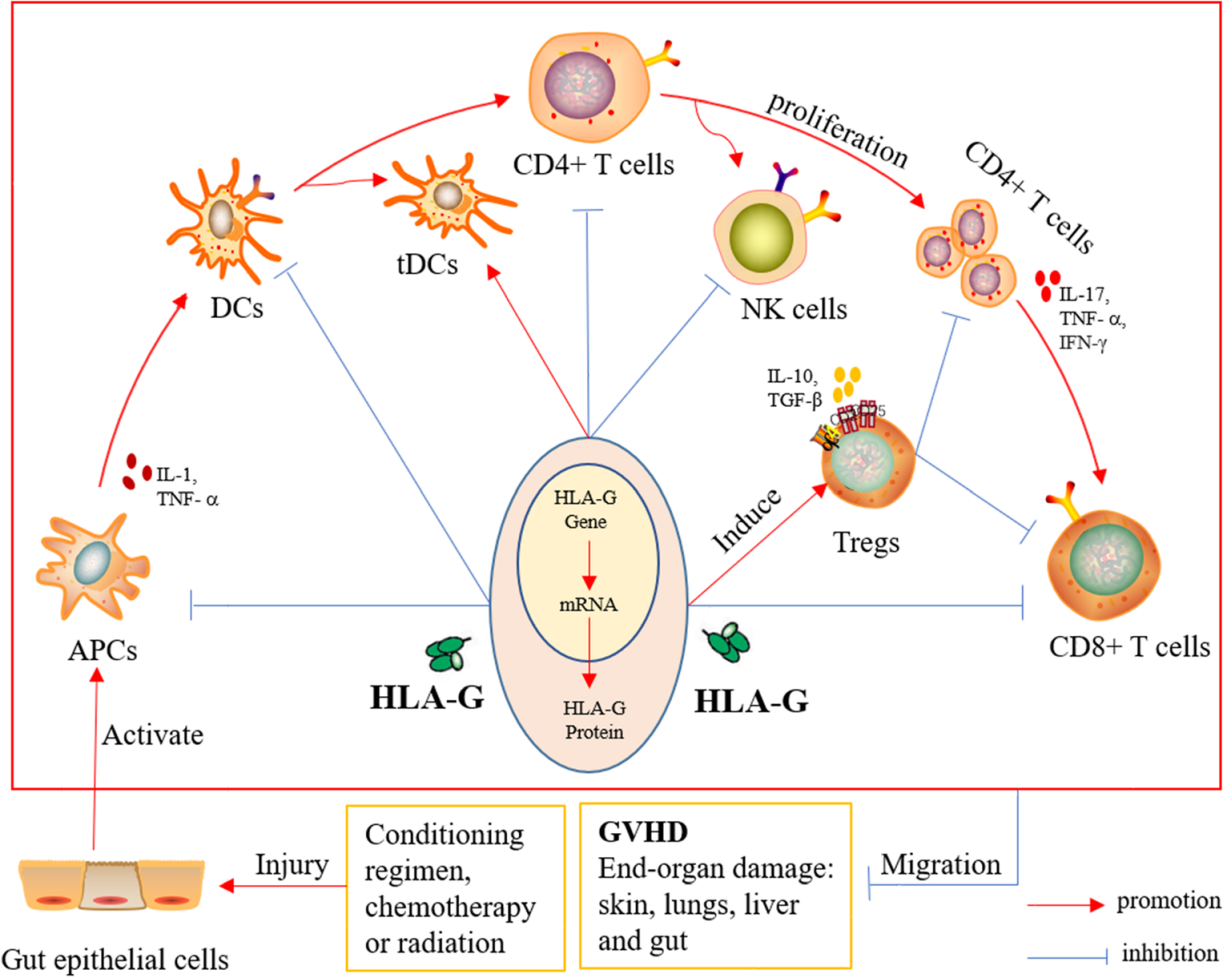
Mechanism of immune regulation of HLA-G in graft-versus-host disease. Red arrows indicate promotion; blue segments indicate inhibition. HLA-G reduces the occurrence of GVHD through direct and indirect regulatory mechanisms. HLA-G can interact with inhibitory receptors, directly inhibiting immune effectors such as T cells, NK cells, and APCs. On the other hand, HLA-G induces the production of tolerant dendritic cells and regulatory T cells, thereby causing further inhibition of the effector cells. *APCs* antigen-presenting cells, *DCs* dendritic cells, *tDCs* tolerant dendritic cells, *Tregs* regulatory T cells

HLA-G preferentially serves as a ligand for inhibitory HLA-G receptors to exert its immunomodulatory and immunosuppressive properties involved in transplantation tolerance. Meanwhile, inhibitory HLA-G receptors exist on the surface of many kinds of immune cells, such as natural killer cells that express killer cell immunoglobulin-like inhibitory receptor 2DL4 (KIR2DL4) (also known as CD159d) and immunoglobulin-like transcriptional receptor 2 (ILT-2) (also known as CD85j and LILRB1), lymphocytes and granulocyte monocytes express ILT-2, dendritic cells (DCs), and macrophages and monocytes express ILT-4 (also known as CD85d and LILRB2)[40]. By interacting with its cognate receptors of the above immune cell subsets (Fig. 1), HLA-G is able to effectively inhibit the cytolytic activity of NK cells and CD8+T cells, restrain the allo-proliferative responses of CD4+T cells, block the progress of allogeneic T cells in the cell-cycle, inhibit the production and activation of antigen-presenting cells, suppress the differentiation and proliferation of B cells, as well as reduce the release of antibody and proinflammatory cytokines, and thus, peripheral immune tolerance is achieved after transplantation and the incidence and severity of GVHD are reduced. Furthermore, HLA-G was shown to indirectly regulate the immune system by inducing regulatory T cells (Tregs) and tolerant dendritic cells (tDCs) to demonstrate a long-term immune tolerance [6, 41]. Such a development of tolerance can be extraordinary beneficial to allo-HSCT, decreasing the rate of aGVHD and cGVHD as well as reducing dependency on immune-suppressive drug regiments.

## The role of HLA-G in graft-versus-host disease

### Correlation between HLA-G and GVHD

HLA-G was initially detected on the surface of cytotrophoblast cells at the maternal-fetal interface and exhibits a crucial role in maternal-fetal tolerance during pregnancy [49]. As a molecule with immunosuppressive function, HLA-G plays a significant role in the development of immune tolerance following transplantation, especially in the field of solid organ transplantation [11]. The expression of HLA-G in heart and kidney transplant patients can reduce the occurrence of transplant rejection [50, 51]. Therefore, the immunoregulatory and immunosuppressive activities of HLA-G have been widely concerned in the development of GVHD after hematopoietic stem cell transplantation in patients with hematologic diseases. In 2008, Le Maux et al. [8] analyzed the soluble HLA-G level in 20 peripheral blood stem cell (PSC)–transplanted patients, showing that sHLA-G molecules play a negative role in the development of aGVHD, and the incidence of aGVHD was low in patients with increased HLA-G expression over time after allo-HSCT, involved specifically in aGVHD prevention. Nomura et al. [12] also draw the similar conclusion that elevated HLA-G/sHLA-G participates in the pathophysiology and prevention of aGVHD following HSCT. Liu et al. [9], through examining the sHLA-G5, sHLA-G6, and sHLA-G7 levels in 106 patients with hematological malignancies before transplantation, on days +15 and +30 after transplantation, revealed that the levels of HLA-G5 were significant lower in patients with grade II–IV aGVHD compared to those with grade 0–I aGVHD, confirming that the increased levels of sHLA-G5 after transplantation were negatively correlated with the severity of aGVHD, and notably, sHLA-G5 rather than sHLA-G6 or sHLA-G7 might predict the development and severity of aGVHD. Biedron et al. [10] also reached a similar conclusion that soluble HLA-G level and HLA-G receptors (ILT2, ILT4, and KIR2DL4) are involved in the intensity of aGVHD and might be a predictor of aGVHD. Kordelas et al. [11] showed that elevated soluble HLA-G levels were not only associated with less severe aGVHD but also with a superior overall survival. They also found that sHLA-G levels were positively relevant to the proportion of regulatory T cells in vivo, and patients with ATG-treated conditioning regimens showed remarkably higher levels of sHLA-G, tempting to speculate that ATG treatment improved the level of sHLA-G via IL-10, which in turns led to the induction and expansion of regulatory T cells. In contrast, Waterhouse et al. [52] carried out research on 59 patients undergoing allo-HSCT and found no correlation between sHLA-G concentration and post-transplantation complications such as aGVHD, cGVHD, relapse, or death. The possible explanation for contradictory conclusions may be HLA disparity, gender mismatching, individual factors, disease status before transplantation, the stem cell dose, conditioning regimen, GVHD prophylaxis, and so on.

The specific implications of soluble human leukocyte antigen-G in the development of GVHD are presented in Table 1.

**Table 1:** Results of correlation between HLA-G and GVHD

Study	N	Role in GVHD
Le Maux et al. [8]	20	Soluble HLA-G level was significantly higher before transplantation and post-allograft period of without aGVHD patients compared with aGVHD patients. sHLA-G molecules could be involved in aGVHD prevention
Nomura et al. [12]	135	Elevated HLA-G/sHLA-G participates in the pathophysiology and prevention of aGVHD
Liu et al. [9]	106	The increased levels of sHLA-G5 after transplantation were negatively correlated with the severity of aGVHD. sHLA-G5 might predict the occurrence and severity of aGVHD
Biedron et al. [10]	32	Soluble HLA-G level was related to intensity of GVHD and may play the role of a prognostic factor for the development of GVHD and the clinical course of this reaction
Kordelas et al. [11]	32	Elevated sHLA-G levels were correlated with less severe acute and chronic GvHD and with a superior overall survival
Waterhouse et al. [52]	59	Soluble HLA-G concentration had no correlation between post-transplantation complications such as aGVHD, cGVHD, relapse, or death

### Correlation between 14-bp polymorphism and GVHD

The HLA-G locus is scarcely polymorphic in the coding region, but several genetic variations at the 3′ untranslated region (UTR) have been identified [22]. Previous studies have proposed that the HLA-G mRNA stability and the expression of HLA-G are related to 14-base pair (bp) insertion/deletion (ins/del) polymorphism in exon 8 in the 3′ UTR of the HLA-G gene. Chen et al. [53], through genotyping the14-bp ins/del gene polymorphism and determining the expression of plasma sHLA-G in 150 normal Chinese Han population, found that plasma-soluble HLA-G level with homozygotes for 14-bp del and heterozygotes was dramatically higher than that with the homozygotes for 14-bp ins, whereas there was no remarkable difference between the homozygotes for 14-bp del and heterozygotes. The same results have been proved in Brazilian and French population [54], and consensually, the 14-bp ins/ins genotype leads to lower HLA-G protein expression [55]. Taking these into consideration, it seems to be implicated that the 14-bp polymorphisms that favor HLA-G expression must be linked to less severe GVHD. In 2011, Boukouaci et al. [56] analyzed HLA-G 14-bp ins/del dimorphism in 157 patients with sibling bone marrow transplantation, in which univariate analysis showed that the homozygotes for 14-bp insertion was correlated with the occurrence of severe aGVHD (grade III and IV), and multivariate analysis further confirmed the correlation, suggesting that the decrease of HLA-G expression caused by 14-bp insertion is a risk factor of severe aGVHD. Other studies have drawn different conclusions. La Nasa et al. [57] investigated 14-bp polymorphism in 53 unrelated donor HSCT for thalassemia and found that homozygote for 14-bp deletion had a higher risk of aGVHD than homozygote for14-bp insertion. Sizzano et al. [58] did not find that 14-bp ins/ins homozygous patients had a higher risk of severe aGVHD than 14-bp del/del homozygous and 14-bp ins/del heterozygous patients. Waterhouse et al. [52] proved that 14-bp ins/ins patients showed remarkable lower pre-transplantation levels of HLA-G compared to those carrying the 14-bp del/del and 14-bp ins/del genotype, but there was no significant difference between the 3 different genotype groups after HSCT, and the recipients or donor HLA-G 14-bp polymorphism was not associated with the occurrence of aGVHD, cGVHD, relapse, or death. Although there was no association between 14-bp genotype polymorphisms and aGVHD, Chiusolo et al. [59] observed that 14-bp ins/del and 14-bp del/del patients were characterized by a higher OS and DFS. The reasons for the controversial results about the correlation between14-bp polymorphism and GVHD are as follows: firstly, HLA-G molecules have ethnical differences; secondly, the incidence of HLA-G 14-bp polymorphisms in the general population is different; thirdly, the HLA system is complex; fourthly, the screening conditions of each research center are different; and last but not least, a large cohort of patients should be performed to further confirm the present results. Therefore, further studies need to be performed to investigate whether HLA-G polymorphism could result in the occurrence of GVHD by affecting the level of HLA-G.

The specific implications of 14-bp polymorphism in the occurrence of GVHD are listed in Table 2.

**Table 2:** Results of correlation between 14-bp polymorphism and GVHD

Study	N	Role in GVHD
Boukouaci et al. [56]	157	The HLA-G low expressor 14bp ins allele constituted a risk factor for the incidence of severe aGVHD
La Nasa et al. [57]	53	Homozygous for the 14-bp deletion had a higher risk of developing aGVHD than homozygous for the 14-bp insertion. The 14-bp polymorphism could be an important predictive factor for aGVHD
Sizzano et al. [58]	147	The 14-bp ins had no association between graft rejection, TFS, OS, or PMC
Waterhouse et al. [52]	59	HLA-G 14-bp polymorphism had no correlation between post-transplantation complications such as aGVHD, cGVHD, relapse, or death
Chiusolo et al. [59]	47	HLA-G 14-bp polymorphism had no correlation between the risks of aGVHD occurrence. The 14-bp ins/14-bp ins genotype was characterized by a lower OS and DFS

*TFS* thalassemia-free survival, *OS* overall survival, *PMC* persistent mixed chimerism

## HLA-G as a potential biologic immunosuppressant for GVHD

Mesenchymal stem cells (MSC) are adult stem cells that exist in many kinds of tissues, such as bone marrow, umbilical cord placenta, and adipose tissue, which have the functions of multi-directional differentiation, immune regulation, supporting hematopoiesis, and tissue repair and have been extensively used in the treatment of regenerative medicine and autoimmune diseases. Since Le Blanc et al. [60] first reported the striking results of MSC used in a patient with severe treatment-resistant grade IV aGVHD, MSC has become an alternative cell therapy, which is commonly used in the second-line treatment of aGVHD [61]. Studies have observed that HLA-G5 was expressed in mesenchymal stem cells, and it mediated the modulation of immune response by MSC [41, 62]. Moreover, data showed that MSC^HLA-G+^ had better immunosuppressive functions than do MSC^HLA-G+/-^, enhancing inhibition of allogeneic T cell proliferation in vitro. The above data are in favor of the potential application of HLA-G5 combined with MSC as immunosuppressant cells to treat aGVHD. Kordelas et al. [63] demonstrated that the application of MSC-derived exosomes containing high concentrations of HLA-G in a patient with steroid-refractory severe acute GVHD has led to a remarkable improvement in GVHD symptoms. Collectively, HLA-G could be a specific marker for therapeutic approaches using MSCs or MSC-derived exosomes to treat therapy-refractory aGVHD.

It is widely recognized that regulatory T cells immunotherapy can suppress exuberant immune system activation and promote immunologic tolerance. Numerous studies have shown that Tregs can be used to the prevention and treatment of aGVHD. Edinger et al. [64] showed that adoptive infusion of Tregs after allo-HSCT can prevent the occurrence of aGVHD, which is helpful to immune reconstruction mediated by conventional T cells after transplantation and, meanwhile, retain the graft-versus-leukemia (GVL) effect [65]. However, the low numbers of Tregs in the circulation and Treg stability would affect the therapeutic effect [65, 66]. Stamou et al. [65] exposed human peripheral blood T cells to azacytidine/decitabine to induce the generation of CD4+HLA-G+FOXP3-T cells, which was a stable Treg subset and showed potent suppression function in vitro. Taking these into account, it could be hypothesized that hypomethylating agents-induced CD4+HLA-G+FOXP3-T cells in vitro would be adoptive cellular immunotherapy against acute graft-versus-host disease. On the other hand, Favier et al. [67] injected with B2M-associated HLA-G5-coated beads into skin allograft recipient mice and demonstrated the tolerogenic function of B2M-HLA-G5 dimers in the context of skin allograft transplantation. LeMaoult et al. [68] also showed that synthetic HLA-G proteins could be therapeutic use in transplantation.

For the above available data along with the proved transplantation tolerance induced by HLA-G and the relationship between HLA-G levels and GVHD, therapeutic HLA-G molecules have been proposed as a promising and ideal biologic immunosuppression candidate for clinical use to the prevention and treatment of GVHD. However, with the problems of structure and lack of a stable purified molecule, the clinical use of HLA-G molecules as therapeutic agents faces several hindrances. Future development will optimize the synthesis of stable HLA-G proteins and then evaluate its ability in vivo as a recombinant molecule alone or in combination with immunosuppressant drugs to prevent and/or treat GVHD. In addition, the use of mesenchymal stem cells or regulatory T cells that express or secrete HLA-G to prevent and/or treat GVHD is a promising strategy in the future, which could improve the clinical outcomes of patients.

## Conclusion and perspectives

Graft-versus-host disease is the most common serious complication after allogeneic hematopoietic stem cell transplantation. Revealing the mechanism of GVHD and then guiding the prevention even the treatment of GVHD has become widely concerned and studied. Studies have confirmed that HLA-G can directly interact with immune cells and inhibit the cellular and humoral immune function of the body, thus contributing to induce immune tolerance and reduce the incidence of GVHD. Continuing and in-depth understanding the role of HLA-G in immune regulation and in the context of graft-versus host disease could not only develop the basic theory of immunology but also have the potential to open up new avenues for clinical diagnosis, prognosis evaluation, as well as providing a new direction for the treatment of GVHD.
